# Methodological approaches for identifying competencies for the physiotherapy profession: a scoping review

**DOI:** 10.1007/s44217-022-00008-9

**Published:** 2022-06-28

**Authors:** Stephanie Scodras, Kyla Alsbury-Nealy, Heather Colquhoun, Euson Yeung, Susan B. Jaglal, Nancy M. Salbach

**Affiliations:** 1 Temerty Faculty of Medicine, Rehabilitation Sciences Institute, University of Toronto, 500 University Avenue, Suite 160, Toronto, ON M5G 1V7 Canada; 2 Department of Occupational Science and Occupational Therapy, Temerty Faculty of Medicine, University of Toronto, Toronto, ON Canada; 3 Department of Physical Therapy, Temerty Faculty of Medicine, University of Toronto, Toronto, ON Canada; 4 University Health Network, KITE Research Institute, Toronto, ON Canada

**Keywords:** Clinical competence, Professional competence, Competency-based education, Physical therapists, Physical therapy specialty

## Abstract

Physiotherapy competencies inform the education and regulation of the profession. Many different methods appear to be used to identify competencies and there is no consensus on optimal methods to identify competencies. The purpose of this review is to synthesize the methodological approaches used to identify competencies for the physiotherapy profession and summarize the nature of those competencies. We searched MEDLINE, EMBASE, CINAHL, and the grey literature from inception to June 2020. Two independent reviewers screened for empirical peer-reviewed articles that aimed to identify professional physiotherapy competencies. General study characteristics, competency characteristics (e.g., target practice area), and methodological characteristics (e.g., study population, data collection and analysis method for each methodological step) were extracted. Descriptive statistics and narrative synthesis were performed. Of the 9529 references screened, 38 articles describing 35 studies published between 1980 and 2020 were included. Orthopaedics (20.0%) was the most commonly targeted area of practice. Studies used one to eight methodological steps whose objective was to generate (16 studies), validate (18 studies), assign value (21 studies), refine (10 studies), or triangulate (3 studies) competencies, or to address multiple objectives (10 studies). The most commonly used methods were surveys to assign value (n = 20, 95%), and group techniques to refine competencies (n = 7, 70%). Physiotherapists with experience in the area of competence was the most commonly consulted stakeholder group (80% of studies). This review can provide methodological guidance to stakeholders such as educators and regulators that aim to identify professional competencies in the future.

**Supplementary Information:**

The online version contains supplementary material available at 10.1007/s44217-022-00008-9.

## Introduction

Competence, defined as “the habitual and judicious use of communication, knowledge, technical skills, clinical reasoning, emotions, values, and reflection in daily practice for the benefit of the individual and the community being served” [1], is an important concept for healthcare professionals. Competence is essential for providing appropriate and optimal patient care [2]. It is considered task-specific, meaning that competence in one area of practice does not necessarily translate to another [3]. Therefore, to develop holistically competent practitioners, it is important to explore what constitutes competence for the breadth of tasks that healthcare professionals must perform. This leads to the study of specific competencies, which are the building blocks of competence.

Competencies are the trainable and measurable knowledge, skills, and attitudes required to perform professional activities [4, 5]. Identifying competencies informs the development of entry-level and post-graduate curricula and training in several healthcare professions such as medicine, nursing, and physiotherapy [6–8]. It also informs the assessment and regulation of healthcare professionals to ensure safe and competent practice [9–11].

While the concepts of competence and competency-based education and assessment have been comprehensively explored in medicine [6], they have received less attention in physiotherapy [8]. Physiotherapy plays an essential role in the healthcare system through the rehabilitation and management of injury, disease, and chronic conditions that affect all body systems [12, 13]. The discipline is expanding as the scope of practice increases and physiotherapists fill emerging clinical and non-clinical roles [14–16]. Therefore, it is important for physiotherapists to be competent in a variety of professional activities. Although a number of studies to identify physiotherapy competencies have been conducted, the optimal methodological approach is unknown. Synthesizing the types of physiotherapy competencies that have been the target of research and the methods used to identify them may provide guidance for the design and implementation of future research in this area, and serve as a first step in developing best practices. It may also provide a framework for stakeholders interested in identifying competencies for other healthcare professions.

Currently, literature on methods for identifying competencies for physiotherapists is limited. A recent scoping review that synthesized methods used in peer-reviewed and grey literature that produced competency frameworks found that there was substantial variability in methodological approaches for developing competency frameworks between and within healthcare profession-specific literature [17]. The authors also found that the majority of competency framework-development studies employed a multi-step approach and used multiple methods within the same study [17], implying methodological complexity in this area of research. However, their summary of existing guidance for the development of competency frameworks reveals that several sources frame ‘identifying competencies’ as a single step within the framework-development process [17]. This raises the question of how to go about this competency identification step. Furthermore, while their analysis detailed the various outcomes and applications of the methods, it lacked a systematic approach to categorizing the objectives of each methodological step and the types of stakeholders consulted in the research. Additionally, the article did not report the order in which the methods were used, which could benefit future researchers. Finally, the systematic search only identified ten (5%) frameworks that were specific to allied health, which grouped physiotherapy with other disciplines despite the noted variability of methodologies used within profession-specific literature [17]. Therefore, the purpose of this study was to synthesize the nature of competencies identified for the physiotherapy profession and the methodological approaches used to identify those competencies.

## Methods

A scoping review based on the Arksey and O’Malley [18] framework (enhanced by Levac et al. [19]) and the Preferred Reporting Items for Systematic reviews and Meta-Analyses extension for Scoping Reviews (PRISMA-ScR) [20] was conducted. One author (SS) developed the research protocol that was reviewed by a second author (NMS) prior to starting the study. The research protocol was registered with Open Science Framework [21] after the study had begun, however no changes were made to the protocol prior to registration.

### Data sources and searches

A systematic search was conducted in June 2020. Tailored strategies for searching MEDLINE, EMBASE, and CINAHL that combined the main concepts of competency and physiotherapy, with no restrictions on publication year or type, were developed with input from an academic librarian (see Additional File 1 for MEDLINE search). These databases were selected because of their relevance to physiotherapy. A targeted search of the grey literature was also conducted using Google, Scopus, and Proquest Theses and Dissertations Global search engines in September 2020 to identify peer-reviewed research articles. Reference lists of the included articles were scanned. References were managed in EndNote X8 (Clarivate Analytics, Philadelphia, PA).

### Study selection

Studies meeting the following criteria were selected: (i) the article was written in English, (ii) the publication type was an empirical peer-reviewed journal article, (iii) the stated purpose of the study was to identify professional competencies, and (iv) the competencies were targeted to physiotherapy professionals. Empirical peer-reviewed journal articles were the focus of this review because of the level of methodological detail required for publication. Initially, we planned to include articles targeting multi- or inter-professional competencies. During the full-text screening phase, the research team decided to focus the review on physiotherapy competencies only to enable comparison of methodologies.

Using Covidence software (Veritas Health Innovation, Melbourne, Australia), two reviewers (authors SS and KAN) independently piloted the title and abstract screening procedure on 20 articles. They then met to discuss discrepancies and improve the clarity of the screening form. The same two reviewers independently screened titles and abstracts, and, subsequently, full texts. Disagreements were managed by discussion, and discrepancies were discussed with a third researcher (NMS). Final decisions were made by the first author (SS).

### Data extraction

We conducted two steps to extract the data: (1) extracting text verbatim, and (2) categorizing the extracted text. In this first step, data related to general study characteristics, competency characteristics, and methodological characteristics were extracted verbatim to improve transparency and reproducibility. Study characteristics included title, lead author, year, country of origin, study purpose, funding, collaborating professional organization (defined as an organization that assists in the research design or that mandates the study), and target population/application of the findings. Competency characteristics included the target area of practice, type, and target of the competencies. Methodological characteristics included the stated methodology, study population, data collection and analysis methods. If the study used multiple methodological steps, then methodological characteristics were extracted for each step. Two reviewers (SS and KAN) independently piloted the data extraction tool on five publications, and met to review the results, resolve discrepancies through discussion, and subsequently revise the tool. A single reviewer (SS) used REDCap [22, 23] to complete this step for all included publications.

The second step involved creating and operationally defining meaningful categories for each variable to help synthesize the findings. Table 1 presents the variables, categories and associated definitions for study, competency, and methodological characteristics. The categories for physiotherapy area (e.g., orthopaedic, acute care) and method types were developed prior to categorizing the data. They were based on special interest groups of professional associations [24–26] and the method types defined in a recent scoping review [17], respectively. The remaining categories for study purpose, type of competency, stakeholder group consulted, and methodological step objective were developed by two researchers (SS and NMS) based on the extracted text. Methodological step objectives were characterized to facilitate the synthesis of the methodological approaches, which were varied and often involved multiple steps.

**Table 1:** Competency and methodological codes and definitions/descriptions

Variable	Categories	Definition/description
Study purpose	Identify new competencies	The study aims to identify professional competencies for a specific target area of practice or target professional group (i.e., entry-level, practicing, or specialist physiotherapists) and does not use an existing set of competencies as the basis for the study
	Adapt competencies to current context	The study aims to understand the relevance of existing competencies for a different or more specific target population than what the competencies were initially developed for (e.g., competencies identified for physiotherapists in a different country)
	Update/revalidate competencies	Studies that explicitly aimed to update or revalidate a set of competencies for the same target population of physiotherapists
Type of competency	Clinical competencies	Competencies relating to physiotherapy clinical roles (e.g., the assessment and treatment of patients including treating certain conditions, clinical practice in a certain setting)
	Non-clinical competencies	Competencies relating to non-clinical physiotherapy roles (e.g., providing clinical education to physiotherapy students, education program administration, management)
	Clinical and non-clinical competencies	Competencies for physiotherapy clinical and non-clinical roles
Method type	Consensus method	A formal type of method that involves obtaining the input of individuals in order to gain consensus or agreement on competencies or importance of competencies
	Delphi	The Delphi technique is an approach to gather information and reach consensus through structured ‘rounds’ of questionnaires presented to ‘experts’ or individuals with subject matter knowledge.[77]
	Nominal group technique	A structured consensus-building approach that involves face-to-face meetings with ‘experts’.[78]
	Group technique	A formal group of individuals that was convened for the purpose of the study (e.g., task force, steering committee, national advisory committee, subject matter expert group) that involves discussion and/or interaction between members (e.g., conference calls, conference meeting, drafting a data collection instrument as a group)
	Literature review	A method that seeks to identify and synthesize information from published peer-reviewed or non-peer reviewed sources (e.g., a literature search performed to identify relevant literature)
	Mapping exercise	A method used to compare and/or align findings with existing documents (e.g., to ensure alignment with current legislation)
	Qualitative method	Types of methods that involve collecting and analysing non-numerical data (e.g., language, text) within a study that typically aims to “understand more about a phenomenon, rather than 'measure' it.” [79]
	Focus group	Qualitative method to collect data from multiple participants at the same time, generally through guided group discussion
	Interview	Qualitative method to collect data from a single participant at a time using a one-on-one structured or semi-structured or unstructured interviewing approach (e.g., formal or informal interviews)
	Research team consultation	Types of methods involving one or more members of the research team (i.e., authors). This may include discussion or decisions made among the researchers
	Stakeholder consultation	A method that seeks input or feedback from an individual or group with specific characteristic(s) (e.g., physiotherapists, experts, managers, etc.). This process does not involve interaction or discussion between stakeholders
	Card sort process	A type of method that aims to understand how stakeholders organize a set of concepts.[80]
	Survey	A method whereby stakeholders complete an online or print questionnaire
Objective of methodological step	Generate competencies	A methodological step that aims to generate novel competencies (e.g., requesting stakeholders to provide a list of possible competencies for practice, searching the literature for potential competencies to populate a survey questionnaire)
	Validate competencies	A methodological step that aims to validate the content of a list of competencies or data collection form. This step may involve obtaining feedback and thus may generate additional competencies; however, it is considered a validation step if feedback is provided on existing competencies or competencies generated in a previous step
	Assign value to competencies	A methodological step that aims to determine or assign quantitative value to competencies to understand what competencies are important/required for the area of physiotherapy being studied
	Refine competencies	A methodological step that aims to review, revise, or finalize a list of competencies that has been validated or assigned value in a previous step
	Triangulation	A methodological step that compares findings from multiple data sources to determine alignment between the data
Stakeholder group consulted	Physiotherapist(s) with experience	Physiotherapist clinicians or non-clinicians with experience in the area of competence
	Physiotherapist expert(s)	Physiotherapist clinicians or non-clinicians deemed ‘experts’ in the area of competence (e.g., labeled as experts by the authors, recruited to a Subject Matter Expert group, specialists), unless belonging to a more specific group below (e.g., entry-level educators, etc.)
	Physiotherapist(s) general	Physiotherapists in general (i.e., not requiring experience in area of physiotherapy being studied)
	Physiotherapist new graduate(s)	Physiotherapists defined by the authors as new graduates
	Physiotherapist clinical educator(s)	Physiotherapists in a clinical educator/instructor role for entry-level physiotherapy students
	Entry-level educator(s)	Individuals responsible for teaching within entry-level physiotherapy programs (stated expert or non-expert)
	Post-graduate educator(s)	Individuals responsible for teaching post-graduate physiotherapy courses (stated expert or non-expert)
	Educator(s) (unspecified)	Individuals responsible for teaching physiotherapists, but not specified between entry-level and post-graduate level (stated expert or non-expert)
	Professional organization(s)	Appointed/elected formal members of a professional physiotherapy organization association (e.g., representative from the American Physical Therapy Association)
	Non-physiotherapist(s) with experience	Individuals who are not physiotherapists with experience in the area of competence (e.g., rheumatologists, human resources development professionals)
	Non-physiotherapist expert(s)	Individuals who are not physiotherapists deemed ‘experts’ (e.g., labeled as experts by the authors, or recruited to a Subject Matter Expert group)
	Interdisciplinary educator(s) or academic administrator(s)	Individuals who are labeled as interdisciplinary educators or administrators (e.g., interdisciplinary program faculty)
	Manager(s) or employer(s)	Individuals responsible for managing and/or employing physiotherapists or physical therapy services (stated expert or non-expert)
	Patient(s) or families	Patients and/or families with experience with the area of competence being studied
	Researchers of the study	Researchers or authors of the study
	Expert(s) (unspecified)	Individuals deemed ‘experts’, but not specified if physiotherapists or non-physiotherapists

Once the categories were developed, they were added to the data extraction form in REDCap in the form of multiple-choice questions with dichotomous or nominal response scales. Adding the categories for the variables of interest to the REDCap data extraction form allowed the categorical data and the verbatim text to be visualized simultaneously. This helped to avoid errors while categorizing the data. A single analyst (SS) categorized the extracted text, and met with another research team member (NMS) regularly to discuss any uncertainties.

### Data synthesis and analysis

The categorical data and verbatim text were exported into Microsoft Excel (v16.0.5227.1000, Microsoft, Redmond, WA, USA) for analysis. Descriptive statistics (frequencies and percentages) were used to summarize categorical data. The data that were extracted verbatim were used to provide examples for the narrative synthesis.

Competency characteristics and stakeholder groups consulted were presented within each targeted professional group and for all groups combined. The research methods used and stakeholder groups consulted across studies were presented based on the objective of the methodological step. Analysing the frequency that each method type was used and each stakeholder group was consulted based on the objective of each step provided a more meaningful comparison between studies. For example, some studies could use similar methods for different reasons (e.g., using a group technique to generate an initial list of possible competencies vs. using a group technique to refine a list of competencies that had already been rated on a scale of importance) but this difference would be lost if the analysis was performed at the level of the methodological approach as a whole rather than the level of the step.

## Results

### Search results and study selection

**Fig. 1:**
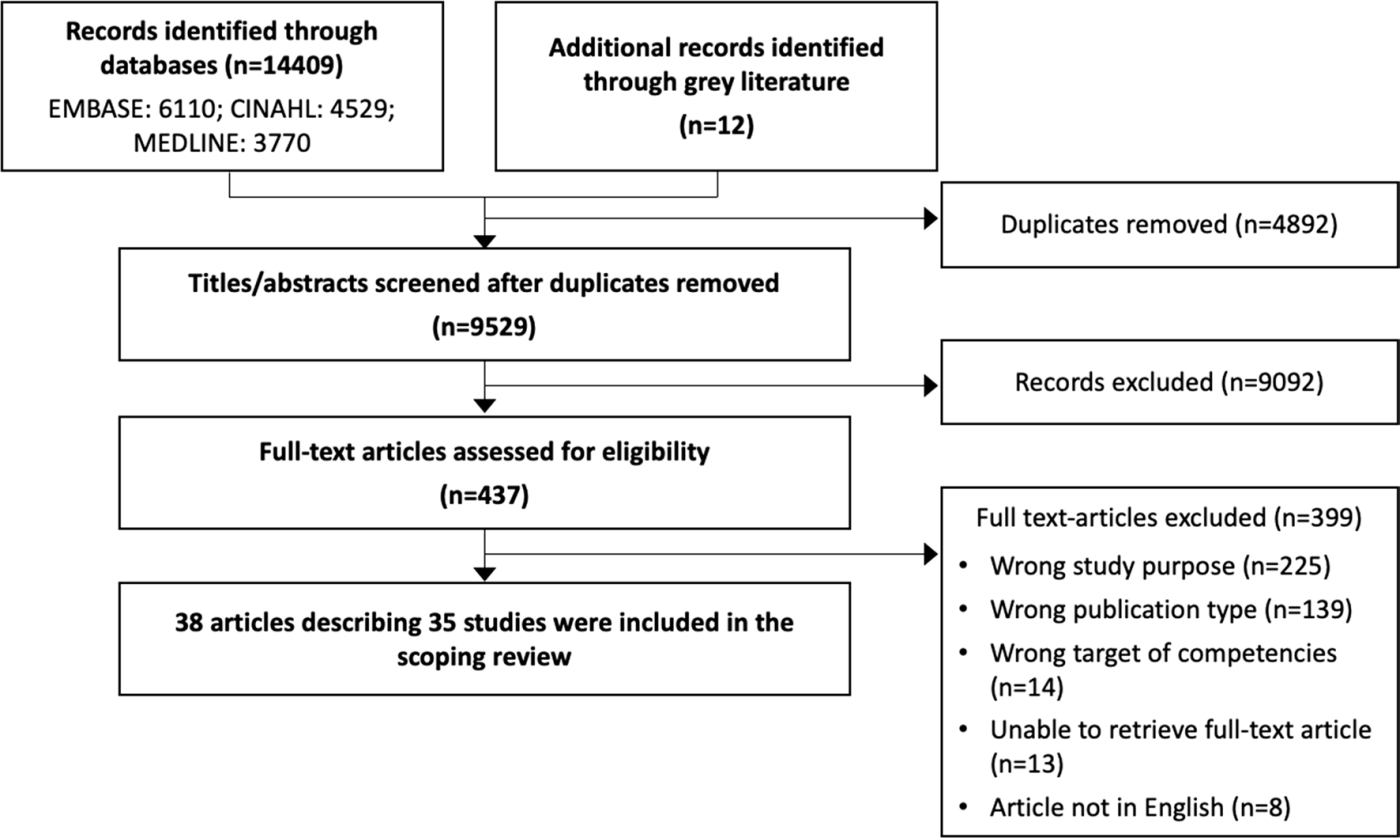
Process of study selection

The search yielded 9529 unique records. After title and abstract screening, we reviewed 437 full texts and included 38 articles describing 35 studies in the review (Fig. 1). None of the records identified in the grey literature were included.

### Characteristics of included studies

Table 2 presents characteristics of included studies. Included studies were published between 1980 and 2020. Of the 35 studies, the majority were conducted in the USA (n = 24, 68.6%), followed by Canada and the United Kingdom (n = 3, 8.6% per country), Australia and South Africa (n = 2, 5.7% per country), and Kuwait (n = 1, 2.8%). Studies aimed to identify new competencies (n = 17, 48.6%) [27–46], adapt existing competencies to a new context of interest (n = 9, 25.7%) [47–55], or update/revalidate competencies (n = 9, 25.7%) [56–64].

**Table 2:** Characteristics of included studies organized by study purpose

	Author, year, country	Collaborating professional organization (if applicable)	Study purpose	Source of competencies	Methodological step(s) Method (objective(s) of step)	Stakeholder group(s) consulted^b,c^	Practice area targeted	Target population for competencies
1	Moncur, 1985, USA^a^/Moncur, 1987, USA^a^		Identify new competencies	Current study	1.Research team consultation (Generate) 2.Stakeholder consultation (Validate) 3.Survey (Assign value)	•Researcher(s) of study •PT expert(s) •Non-PT expert(s) •PT(s) with experience •PT clinical educator(s) •Non-PT(s) with experience	Orthopaedic	Entry-level physiotherapists
2	Donato, 2004, USA^a^	APTA	Identify new competencies	Current study	1.Research team consultation (Generate) 2.Consensus—Delphi (Validate) 3.Survey (Assign value)	•Researcher(s) of study •PT(s) with experience × 2 •PT(s) general	Orthopaedic	Practicing physiotherapists
3	Gamboa, 2005, USA^a^	APTA	Identify new competencies	Current study	1.Literature review (Generate) 2.Group technique (Generate) 3.Group technique (Generate) 4.Survey (Assign value) 5.Group technique (Refine)	•PT expert(s) × 3 •Researcher(s) of study •PT(s) with experience	Orthopaedic	Specialist physiotherapists
4	Sizer, 2007, USA		Identify new competencies	Current study	1.Consensus—Delphi (Generate, Assign value, Refine)	•Entry-level educator(s) •Post-graduate educator(s)	Orthopaedic	Practicing physiotherapists
5	Briggs, 2012, Australia^a^		Identify new competencies	Current study	1.Consensus—Delphi (Generate, Validate, Assign value) 2.Literature review (Generate) 3.Mapping exercise (Triangulate)	•PT(s) with experience •Non-PT(s) with experience •Patient(s) or families	Orthopaedic	Practicing physiotherapists
6	Langridge, 2019, UK		Identify new competencies	Current study	1.Qualitative—Interview(s) (Generate) 2.Qualitative—Focus group(s) (Validate)	•PT expert(s) × 2 •Non-PT(s) with experience	Orthopaedic	Practicing physiotherapists
7	Aston-McCrimmon, 1983, Canada / Aston-McCrimmon, 1986, Canada		Identify new competencies	Current study	1.Research team consultation (Generate) 2.Stakeholder consultation (Validate) 3.Survey (Assign value)	•Researcher(s) of study •PT expert(s) × 2 •PT(s) general •PT new graduate(s)	General professional competencies	Entry-level physiotherapists
8	May, 1983, USA		Identify new competencies	Current study	1.Research team consultation (Generate) 2.Survey (Assign value)	•Researcher(s) of study •PT(s) with experience	General professional competencies	Practicing physiotherapists
9	Forbes, 2018, Australia		Identify new competencies	Current study	1.Consensus—Delphi (Generate, Validate, Assign value)	•PT expert(s)	General professional competencies	Practicing physiotherapists
10	Gorman, 2010, USA^a^	APTA	Identify new competencies	Current study	1.Group technique (Generate) 2.Survey (Assign value) 3.Group technique (Refine)	•PT expert(s) × 2 •Entry-level educator(s) × 2 •Professional organization(s) × 2 •PT(s) with experience	Acute care	Practicing physiotherapists
11	Greenwood, 2017, USA^a^	APTA	Identify new competencies	Current study	1.Group technique (Generate) 2.Stakeholder consultation (Validate) 3.Stakeholder consultation (Refine)	•PT(s) with experience × 2 •PT expert(s) × 2 •Entry-level educator(s) × 2 •Professional organization(s) × 2 •PT clinical educator(s)	Acute care	Entry-level physiotherapists
12	Thow, 2004, UK		Identify new competencies	Current study	1.Group technique (Generate) 2.Survey (Assign value)	•PT(s) with experience × 2	Cardiorespiratory	Practicing physiotherapists
13	Hanekom, 2015, South Africa^a^ / van Aswegen, 2017, South Africa		Identify new competencies	Current study	1.Consensus—Nominal group technique (Generate, Validate, Assign value)	•PT(s) with experience	Cardiorespiratory	Practicing physiotherapists
14	Levangie, 1980, USA^a^		Identify new competencies	Current study	1.Not specified (Generate) 2.Survey (Assign value)	•Not specified •PT(s) with experience •PT expert(s) •PT(s) general	Pediatric	Practicing physiotherapists
15	Bryan, 1993, USA^a^		Identify new competencies	Current study	1.Qualitative—Interview(s) (Generate, Validate) 2.Qualitative—Interview(s) (Generate, Validate)	•PT(s) with experience •Non-PT(s) with experience •Post-graduate educator(s) × 2 •Manager(s) or employer(s) × 2 •PT expert(s) •Non-PT expert(s)	Occupational health	Practicing physiotherapists
16	Brody, 2007, USA	APTA	Identify new competencies	Current study	1.Group technique (Generate) 2.Consensus—Delphi (Validate, Assign value) 3.Group technique (Refine)	•PT expert(s) × 3 •PT(s) with experience	Aquatic therapy	Specialist physiotherapists
17	Magnusson, 2020, USA	APTA	Identify new competencies	Current study	1.Research team consultation (Generate) 2.Consensus—Delphi (Validate, Assign value)	•Researcher(s) of study •PT expert(s) •Entry-level educator(s)	Health promotion and wellness	Entry-level physiotherapists
18	Lopopolo, 2004, USA		Adapt competencies to new context	Existing competencies	1.Group technique (Validate) 2.Consensus—Delphi (Validate, Assign value)	•PT(s) with experience × 2 •Researcher(s) of study	Leadership and management	Entry-level physiotherapists
19	Schafer, 2007, USA^a^		Adapt competencies to new context	Existing competencies	1.Research team consultation (Validate) 2.Stakeholder consultation—Card sort technique (Validate) 3.Research team consultation (Validate) 4.Survey (Assign value)	•Researcher(s) of study × 2 •Expert(s) (not specified if PT or non-PT) •PT(s) with experience •Entry-level educator(s)	Leadership and management	Entry-level physiotherapists
20	Bennie, 2019, USA		Adapt competencies to new context	Existing competencies	1.Stakeholder consultation (Validate) 2.Survey (Assign value)	•PT expert(s) •PT(s) with experience	Leadership and management	Practicing physiotherapists
21	Bryan, 1994, USA		Adapt competencies to new context	Existing competencies	1.Survey (Assign value)	•PT(s) with experience	Occupational health	Practicing physiotherapists
22	Sole, 2000, South Africa		Adapt competencies to new context	Existing competencies	1.Stakeholder consultation (Validate) 2.Survey (Assign value)	•Expert(s) (unspecified) •PT(s) with experience •Non-PT(s) with experience	Sports	Practicing physiotherapists
23	Cassady, 2014, Canada		Adapt competencies to new context	Existing competencies and current study	1.Qualitative—Interview(s) (Generate, Validate)	•PT(s) with experience	Global health	Practicing physiotherapists
24	Al-Sayegh, 2016, Kuwait		Adapt competencies to new context	Existing competencies	1.Stakeholder consultation (Validate) 2.Stakeholder consultation (Validate) 3.Survey (Assign value)	•Experts (unspecified) •PT expert(s) •Non-PT expert(s) •Entry-level educator(s) •PT(s) general	Cardiorespiratory	Practicing physiotherapists
25	Weaver, 2018, USA^a^		Adapt competencies to new context	Existing competencies	1.Survey (Assign value)	•PT(s) with experience	Pediatric	Practicing physiotherapists
26	Twose, 2019, UK		Adapt competencies to new context	Existing competencies	1.Consensus—Delphi (Validate, Assign value)	•PT(s) with experience •Entry-level educator(s)	Acute care	Practicing physiotherapists
27	Zachazewski, 1994, USA	ABPTS	Update/ revalidate competencies	Existing competencies	1.Group technique (Validate) 2.Survey (Assign value) 3.Group technique (Refine)	•PT expert(s) × 3 •PT(s) with experience	Sports	Specialist physiotherapists
28	Weber, 2009, USA	ABPTS	Update/ revalidate competencies	Existing competencies	1.Group technique (Validate) 2.Survey (Assign value) 3.Group technique (Refine)	•PT expert(s) × 3 •Professional organization(s) × 2 •PT(s) with experience	Sports	Specialist physiotherapists
29	Mulligan, 2014, USA	ABPTS	Update/ revalidate competencies	Existing competencies	1.Group technique (Validate) 2.Survey (Assign value) 3.Group technique (Refine)	•PT expert(s) × 3 •Professional organization(s) × 2 •PT(s) with experience	Sports	Specialist physiotherapists
30	Chiarello, 2006, USA^a^		Update/ revalidate competencies	Existing competencies and current study	1.Not specified (Validate) 2.Literature review (Generate) 3.Qualitative—Focus group(s) (Generate) 4.Mapping exercise (Triangulate) 5.Stakeholder consultation (Refine)	•Not specified •Patient(s) or families •Interdisciplinary educators or academic administrators	Pediatric	Practicing physiotherapists
31	Effgen, 2007, USA^a^		Update/ revalidate competencies	Existing competencies and current study	1.Stakeholder consultation (Validate) 2.Literature review (Generate) 3.Qualitative—Focus group(s) (Generate) 4.Mapping exercise (Triangulate) 5.Stakeholder consultation (Refine)	•PT(s) with experience × 3 •Professional organization(s) •Interdisciplinary educators or academic administrators	Pediatric	Practicing physiotherapists
32	Aston-McCrimmon, 1984, Canada		Update/ revalidate competencies	Existing competencies	1.Survey (Assign value)	•PT(s) general	General professional competencies	Practicing physiotherapists
33	Milidonis, 1996, USA^a^	ABPTS	Update/ revalidate competencies	Current study	1.Qualitative—Interview(s) (Generate) 2.Group technique (Validate) 3.Survey (Assign value)	•PT(s) with experience × 2 •PT expert(s) × 2 •Educators (unspecified)	Orthopaedic	Specialist physiotherapists
34	Perry, 2008, USA^a^	ABPTS	Update/ revalidate competencies	Existing competencies	1.Group technique (Validate) 2.Survey (Assign value) 3.Group technique (Refine)	•PT expert(s) × 3 •Professional organization(s) × 2 •PT(s) with experience	Neurosciences	Specialist physiotherapists
35	Brody, 2018, USA^a^	APTA	Update/ revalidate competencies	Existing competencies	1.Stakeholder consultation (Validate) 2.Consensus—Delphi (Assign value)	•PT expert(s) •PT(s) with experience	Aquatic therapy	Specialist physiotherapists

Studies are in order of practice area (greatest to least frequent), and year of publication (oldest to most recent)

*PT* physiotherapist; *APTA* American Physical Therapy Association, *ABPTS* American Board of Physical Therapy Specialists; Articles from the same study are listed together and separated by “/”

^a^Indicates funding declared

^b^Populations that were consulted in more than one step are specified with “x # of steps”

^c^Populations were not considered to be consulted for literature review and mapping methods

### The nature of identified competencies

The majority of studies (n = 21, 60.0%) targeted competencies for practicing physiotherapists in general, followed by specialist (n = 8, 22.9%) and entry-level professionals (n = 6, 17.1%) (Table 3). The most common areas of physiotherapy practice were orthopaedics (e.g., management of rheumatoid arthritis) (n = 7, 20.0%), followed by general professional competencies (e.g., patient education), pediatric, and sports physiotherapy (n = 4, 11.4% per area). Approximately half of the studies (n = 18, 51.4%) identified clinical and non-clinical competencies for physiotherapists, including all eight of the studies identifying competencies for specialist physiotherapists.

**Table 3:** Competency characteristics and target population/application of the findings based on target professional group for the competencies and across all studies

	Entry-level PTs (n = 6)	Practicing PTs (n = 21)	Specialist PTs (n = 8)	Pooled (n = 35)
	n (%)			
**Target area of practice**				
Orthopaedic	1 (16.7)	4 (19.0)	2 (25.0)	7 (20.0)
General professional competencies	1 (16.7)	3 (14.3)	0 (0)	4 (11.4)
Pediatric	0 (0)	4 (19.0)	0 (0)	4 (11.4)
Sports	0 (0)	1 (4.8)	3 (37.5)	4 (11.4)
Acute care	1 (16.7)	2 (9.5)	0 (0)	3 (8.6)
Cardiorespiratory	0 (0)	3 (14.3)	0 (0)	3 (8.6)
Leadership and management	2 (33.3)	1 (4.8)	0 (0)	3 (8.6)
Aquatic therapy	0 (0)	0 (0)	2 (25.0)	2 (5.7)
Occupational health	0 (0)	2 (9.5)	0 (0)	2 (5.7)
Global health	0 (0)	1 (4.8)	0 (0)	1 (2.9)
Health promotion and wellness	1 (16.7)	0 (0)	0 (0)	1 (2.9)
Neurosciences	0 (0)	0 (0)	1 (12.5)	1 (2.9)
**Type of competencies identified**				
Clinical competencies	2 (33.3)	10 (47.6)	0 (0)	12 (34.3)
Non-clinical competencies	2 (33.3)	3 (14.3)	0 (0)	5 (14.3)
Clinical & non-clinical competencies	2 (33.3)	8 (38.1)	8 (100)	18 (51.4)
**Setting specified**				
Yes	1 (16.7)	11 (52.4)	0 (0)	12 (34.3)
No	5 (83.3)	10 (47.6)	8 (100)	23 (65.7)
**Target population/application of competencies**				
Post-graduate education providers or administrators	1 (16.7)	10 (47.6)	5 (62.5)	16 (45.7)
Entry-level education providers and administrators	6 (100)	9 (42.9)	0 (0)	15 (42.9)
Practicing physiotherapists	2 (33.3)	8 (38.1)	4 (50.0)	14 (40.0)
Professional physiotherapy organization	1 (16.7)	1 (4.8)	8 (100)	10 (28.6)
Administrators/managers/employers	1 (16.7)	4 (19.0)	0 (0)	5 (14.3)
New graduate & student physiotherapists	1 (16.7)	0 (0)	0 (0)	1 (2.9)
None specified	0 (0)	6 (28.6)	0 (0)	6 (17.1)

The majority of studies (n = 29, 82.8%) identified one or more intended target population/application for the identified competencies (Table 3). Entry-level education providers and administrators were targeted in all six studies that identified competencies for entry-level physiotherapists (e.g., to inform entry-level curricula). Studies that identified competencies for practicing physiotherapists mainly targeted post-graduate education providers and administrators (n = 10, 47.6%) (e.g., to inform post-graduate course content), entry-level education providers and administrators (n = 9, 42.9%) and practicing physiotherapists (n = 8, 38.1%) (e.g., for self-assessment, direct continuing education). Four (19.0%) of these studies also targeted healthcare administrators/managers/employers (e.g., to inform hiring, in-service training). All eight studies that identified competencies for specialist physiotherapists intended the findings to be used by a professional physiotherapy organization (e.g., American Physical Therapy Association to inform specialty practice credentialing programs or exams).

### Methodological approaches for identifying competencies

Across the 35 studies, 8 (22.9%) used 1 methodological step, 10 (28.6%) used 2 steps, 13 (37.1%) used 3 steps, 1 (2.9%) used 4 steps, and 3 (8.6%) used 5 methodological steps, for a total of 86 methodological steps. Across these 86 steps, the objective of the step was to generate competencies (n = 20 (23.3%) steps conducted across 16 studies), validate competencies (n = 21 (24.4%) steps conducted across 18 studies), assign value to competencies (n = 21 (24.4%) steps conducted across 21 studies), refine competencies (n = 10 (11.6%) steps conducted across 10 studies), triangulation (n = 3 (3.5%) steps across 3 studies), or address multiple objectives (n = 11 (12.8%) steps across 10 studies) (Table 4).

**Table 4:** Methods used and stakeholders consulted based on purpose of methodological step

Objective of step	Method used		Stakeholder groups consulted	
*N* = *# of steps across all studies that addressed this objective*	*n (%) of steps that addressed the objective that used a given method type*		*n (%) of steps that consulted with a given stakeholder type*	
Generate competencies (N = 20)	Group technique Research team consultation Literature review^a^ Qualitative method Focus group(s) Interview(s) Not specified	6 (30.0) 5 (25.0) 4 (20.0) 4 (20.0) 2 (10.0) 2 (10.0) 1 (5.0)	PT expert(s) Researcher(s) of study PT(s) with experience Entry-level PT educator(s) Patient(s) or families Professional organization(s) Not specified	6 (30.0) 6 (30.0) 4 (20.0) 2 (10.0) 1 (5.0) 1 (5.0) 1 (5.0)
Validate competencies (N = 21)	Stakeholder method Stakeholder consultation Card sort technique Group technique Research team consultation Consensus method Delphi technique Qualitative method Focus group(s) Not specified	10 (47.6) 9 (42.9) 1 (4.8) 6 (28.6) 2 (9.5) 1 (4.8) 1 (4.8) 1 (4.8) 1 (4.8) 1 (4.8)	PT expert(s) Professional organization(s) Expert(s) (unspecified) PT(s) with experience Researcher(s) of study Non-PT Expert(s) Non-PT(s) with experience Entry-level educator(s) Educator(s) (unspecified) Not specified	12 (57.3) 4 (19.0) 3 (14.3) 3 (14.3) 3 (14.3) 2 (19.5) 1 (4.8) 1 (4.8) 1 (4.8) 1 (4.8)
Assign value to competencies (N = 21)	Survey Consensus method Delphi technique	20 (95.2) 1 (4.8) 1 (4.8)	PT(s) with experience PT expert(s) PT(s) general Non-PT(s) with experience PT clinical educator(s) PT new graduate(s) Entry-level educator(s)	18 (85.7) 7 (33.3) 5 (23.8) 2 (9.5) 1 (4.8) 1 (4.8) 1 (4.8)
Refine competencies (N = 10)	Group technique Stakeholder method Stakeholder consultation	7 (70.0) 3 (30.0) 3 (30.0)	PT expert(s) Professional organization(s) PT(s) with experience Entry-level educator(s) Interdisciplinary educators or academic administrators PT clinical educator(s)	7 (70.0) 7 (70.0) 3 (30.0) 2 (20.0) 2 (20.0) 1 (10.0)
Triangulation (N = 3)	Mapping technique^a^	3 (100)	N/A	
Multiple objectives (N = 11)	Consensus method Delphi technique Nominal group technique Qualitative method Interview(s)	8 (72.7) 7 (63.6) 1 (9.1) 3 (27.3) 3 (27.3)	PT(s) with experience PT expert(s) Entry-level educator(s) Post-graduate educator(s) Non-PT(s) with experience Manager(s) or employer(s) Non-PT expert(s) Patient(s) or families	7 (63.6) 4 (36.4) 3 (27.3) 3 (27.3) 2 (18.2) 2 (18.2) 1 (9.1) 1 (9.1)

*PT* physiotherapist

^a^Stakeholders were not considered to be consulted for literature review and mapping methods

#### Generating and sources of competencies

Across the 20 steps taken to generate a list of novel competencies, group techniques (n = 6, 30.0%) and researcher consultations (n = 5, 25.0%) were the most common methods used, followed by literature reviews and qualitative methods (n = 4, 20.0% per method) (Table 4). Among the competency generation steps, physiotherapist experts and researchers of the studies were the most frequently consulted stakeholder groups (n = 6, 30.0% per stakeholder group). Professional organizations and patients or families were the least frequently consulted groups (n = 1, 5.0% per stakeholder group).

Seventeen studies (48.6%) used an existing set or list of competencies as a starting point for the research (Table 2). The sources of these competencies were varied and included previous research or professional documents that were used to understand which competencies were important for a specific role or professional group (e.g., specific clinical role or new graduates) [49–53, 55], existing competencies for physiotherapists in another country to understand which competencies are important in the country of interest [47, 54], or from existing competencies for other professions to understand the relevance to physiotherapy [48]. Additionally, professional organization documents (e.g., American Physical Therapy Association description of specialty practice or policy statement to be updated) [57–59, 61–63] or results from a previous study on the same topic [56, 64] were sources of competencies used to update/revalidate competencies for the physiotherapy profession.

#### Validating competencies

Across the 17 studies that began with an existing set of competencies, 12 studies (70.6%) used one or more steps to validate those competencies. Overall, across the 21 steps that aimed to validate competencies, stakeholder methods (n = 10 steps, 47.6%) were used most frequently used, and experts (physiotherapists, non-physiotherapists, or unspecified) were the most frequently consulted group of stakeholders in validation steps (n = 17 steps, 81.0%) (Table 4).

#### Assigning value to competencies

Of the 21 steps that aimed to assign value to competencies, surveys were the most frequently used method (n = 20, 95.2%) (Table 4). Assigning value to competencies was primarily achieved by having participants rate the competencies on one or more rating scales (n = 19 steps, 90.5%). Of the 19 steps that used rating scales, the majority (n = 17, 89.5%) included a rating of importance. Eight (47.1%) rating scales of importance were a 5-point scale, five (29.4%) were a 4-point scale, and the rest (4 steps, 23.5%) used 6-, 7-, or 8-point scales. The next most common rating scales were level/extent of knowledge (e.g., minimal, substantial; recall, application) and/or skill (e.g., ability to perform with supervision, independently, etc.) (n = 8 steps, 42.1%) and frequency of use (i.e., how often participants use the competencies) (n = 6 steps, 31.6%). The two surveys that did not assign a value to competencies using a rating scale had participants order a list of competencies in order from most to least important [39] and select important competencies from a list [41]. Physiotherapists with experience in the area of competence (n = 18 steps, 85.7%) were most frequently consulted to assign value to competencies (Table 4).

#### Refining competencies

Across the 10 steps used to refine competencies, group techniques (n = 7,70.0%) were the most frequently used, followed by stakeholder consultations (n = 3, 30.0%) (Table 4). Physiotherapist experts and professional organizations (n = 7 steps, 70.0% per stakeholder group) were the most frequently consulted stakeholder groups for this objective.

#### Triangulation

Triangulation was achieved through mapping exercises (n = 3 steps, 100%). Two studies that employed similar approaches mapped findings from three initial methodological steps to produce a final list of competencies [58, 59]. The other study linked findings from a Delphi step with clinical practice guidelines [29].

#### Methodological steps used for multiple objectives

There were 11 methodological steps that addressed multiple objectives, including three (27.3%) qualitative methods and eight (72.7%) consensus methods. The Delphi techniques either used two [30, 33] or three rounds of surveys [29, 40, 44, 51, 54]. Generating competencies was accomplished by making the first-round questionnaire an open-ended question to elicit responses from the participants regarding important or required competencies for the area of interest. Consensus methods validated competencies by eliciting feedback from participants on the competencies generated in the first round [29, 33, 37, 46] or the competencies that were sourced or generated outside of the consensus method [30, 40, 51, 54].

All seven of the Delphi techniques used a rating scale to assign value to competencies. Similar to the steps that exclusively aimed to assign value to competencies, the majority of the Delphi techniques (n = 6 steps, 85.7%) used a rating scale of importance. Of these scales, three (50.0%) were a 5-point scale, two (33.3%) were a 4-point scale, and one (16.7%) was a dichotomous scale. The next most common rating scales were level/extent of knowledge and/or skill (n = 3 steps, 42.9%). The nominal group technique determined the value of competencies using a ranking process [37, 46].

Regardless of methodological step objective(s), eight (80.0%) of the 10 consensus methods provided a definition for consensus. These were: 75% agreement of participants on a specific cut-off value for one or more rating scales [30, 44, 57]; 70% agreement [37, 46, 54]; 80% agreement [29, 33]; and a cut-off median value as well as 80% agreement of participants [40].

Across the 11 steps that addressed multiple objectives, physiotherapists with experience (n = 7, 63.6%) were the most frequently consulted. Patients or families (n = 1, 9.1%) were among the least frequently consulted groups (Table 4).

#### Stakeholders consulted based on target professional group

Across all 35 studies, physiotherapists with experience in the area of competence (n = 28, 80.0%) and physiotherapist experts (n = 19, 54.3%) were the most frequently consulted stakeholder groups (Table 5). Additionally, physiotherapists with experience in the area of competence were consulted in the majority of studies targeting entry-level physiotherapists (n = 4, 66.7% of 6 studies), practicing physiotherapists (n = 16, 76.2% of 21 studies), and specialist physiotherapists (n = 8, 100% of 8 studies).

**Table 5:** Stakeholder groups consulted based on target professional group for the competencies and across all studies

Stakeholder group	Entry-level PTs (N = 6)	Practicing PTs (N = 21)	Specialist PTs (N = 8)	Pooled (N = 35)
	n (%)			
PT(s) with experience	4 (66.7)	16 (76.2)	8 (100)	28 (80.0)
PT expert(s)	4 (66.7)	7 (33.3)	8 (100)	19 (54.3)
Researcher(s) of study	5 (83.3)	2 (9.5)	1 (12.5)	8 (22.9)
Entry-level educator(s)	3 (50.0)	4 (19.0)	0 (0)	7 (20.0)
Professional organization(s)	1 (16.7)	3 (14.3)	3 (37.5)	7 (20.0)
PT(s) general	1 (16.7)	4 (19.0)	0 (0)	5 (14.3)
Non-PT(s) with experience	1 (16.7)	4 (19.0)	0 (0)	5 (14.3)
Non-PT expert(s)	1 (16.7)	2 (9.5)	0 (0)	3 (8.6)
Expert(s) (not specified if PT or not)	1 (16.7)	2 (9.5)	0 (0)	3 (8.6)
PT clinical educator(s)	2 (33.3)	0 (0)	0 (0)	2 (5.7)
Post-graduate educator(s)	0 (0)	2 (9.5)	0 (0)	2 (5.7)
Interdisciplinary educators or academic administrators	0 (0)	2 (9.5)	0 (0)	2 (5.7)
Patient(s) or families	0 (0)	2 (9.5)	0 (0)	2 (5.7)
PT new graduate(s)	1 (16.7)	0 (0)	0 (0)	1 (2.9)
Manager(s) or employer(s)	0 (0)	1 (4.8)	0 (0)	1 (2.9)
Educators (not specified if entry-level or post-graduate)	0 (0)	0 (0)	1 (12.5)	1 (2.9)
Not specified	0 (0)	2 (9.5)	0 (0)	2 (5.7)

*PT* physiotherapist

**Fig. 2:**
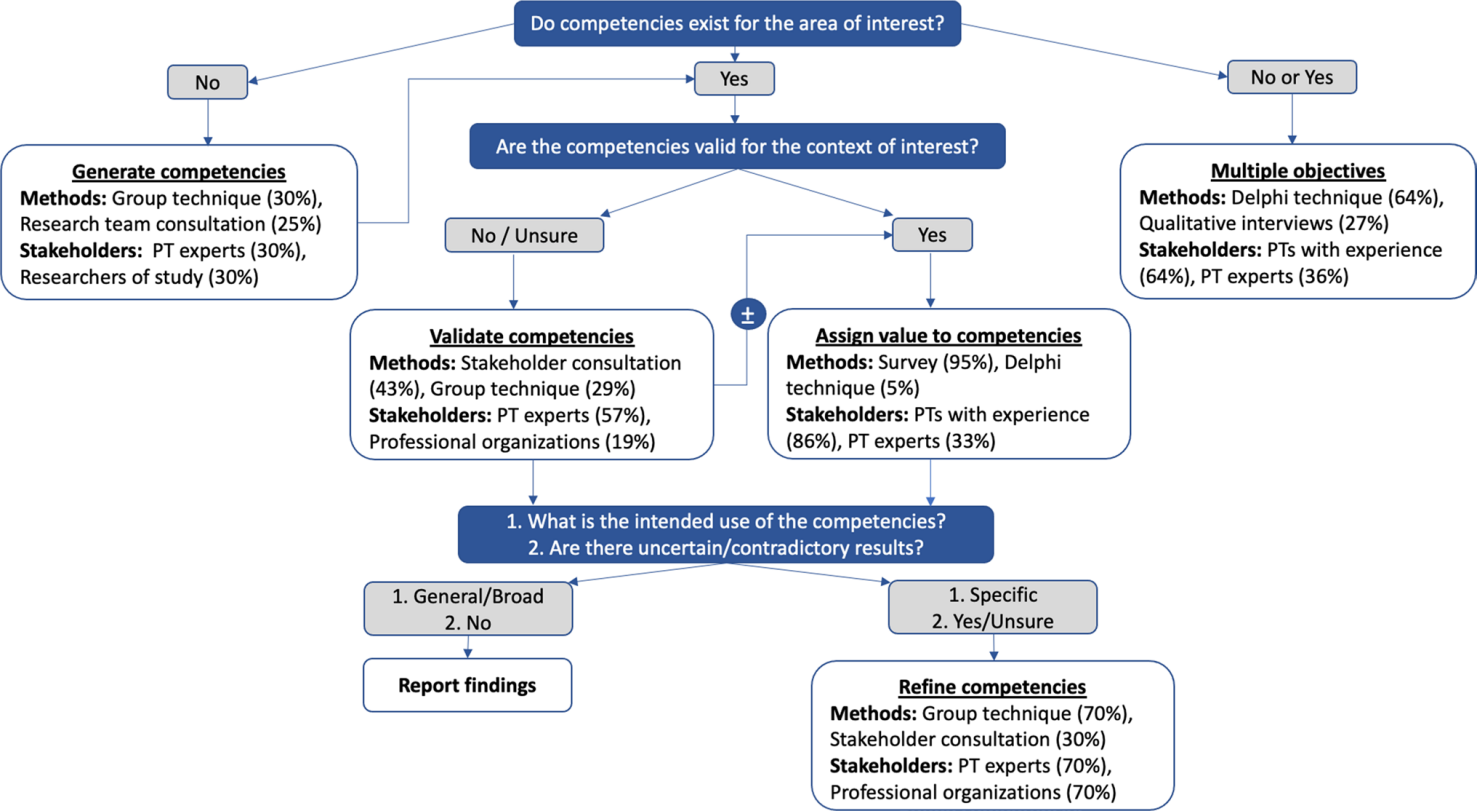
Algorithm of the methods and stakeholders most frequently used to identify physiotherapy competencies by the objective of the methodological step. *PT* physiotherapist

Figure 2 presents an algorithm that summarizes the two most frequently used methods and consulted stakeholder groups for unique objectives relevant to competency identification (e.g., generate competencies, validate competencies, etc.) based on studies included in this review.

## Discussion

This scoping review provides an overview of peer-reviewed empirical studies aiming to identify competencies for the physiotherapy profession. The majority of the studies targeted competencies for practicing physiotherapists and identified clinical and non-clinical types of competencies. Researchers used a variety of methodological approaches, and over three quarters of the studies employed multiple methodological steps to identify competencies. Group techniques and consultations with researchers and stakeholders were the most frequently used methods to generate, validate, and refine competencies, whereas surveys were most frequently used to assign value to competencies. The majority of formal consensus methods that were employed addressed multiple objectives within a single methodological step. Many types of stakeholders were consulted in the competency identification process, and this appeared to vary based on the target professional group.

### The nature of identified competencies

Most studies aimed to identify competencies for practicing physiotherapists. Results from those studies were intended to be used by educators for curriculum development as well as practicing physiotherapists for self-assessment of competence. This finding is in line with the focus on competency-based education approaches in healthcare practitioner training programs that aim to instill the knowledge, skills, and attitudes required for learners to transition successfully into the diverse professional practice roles and settings [5, 7]. Beyond entry-level training, physiotherapists must also remain current in their area of practice and engage in self-reflection to improve practice [65].

Just over half of the studies identified non-clinical competencies, such as the ability to provide clinical education, in addition to clinical ones. This may represent the multi-faceted professional responsibilities of physiotherapists, even in clinical settings. Clinical education is considered to be critical for student development [66], and practitioners recognize their responsibility to provide clinical instruction despite barriers and challenges associated with this role [67].

The most frequently targeted areas for competencies were orthopaedics, followed by general professional practice, pediatrics, and sports. This may be because the majority of physiotherapists work in the areas of musculoskeletal and general practice [12], and several countries recognize speciality areas of practice in pediatrics and sports [68, 69].

### Methodological approaches for identifying competencies

Identifying competencies was often accomplished using several methodological steps and various types of methods. This was an interesting finding given that identifying competencies has been previously framed as a single step [3, 17]. This review also identified five distinct methodological objectives that have been used in various combinations to identify competencies for the physiotherapy profession, namely to generate, validate, assign value, refine, and triangulate competencies.

Of the methods that were employed, group techniques, and stakeholder and research team consultations were the most frequently used to generate, validate, and refine competencies. These methods involve input, feedback, or discussion among stakeholders, which suggests that professional opinions were considered a valuable source of knowledge in competency identification. Interestingly, group techniques were used more frequently than stakeholder consultations. Selecting a group technique that involves discussion among stakeholders, compared to a stakeholder consultation that seeks input from individuals may be influenced by access to certain resources or stakeholders. For example, time constraints and high workloads are common barriers for healthcare practitioners to engage in research, while saliency of the research is a facilitator [70, 71]. Engaging stakeholders that are invested in the research may overcome the obstacles of finding a time and place (in-person or virtually) to bring stakeholders together.

Researchers also sought the opinions of stakeholders to assign numerical value to competencies, and surveys were used more frequently than Delphi techniques for this purpose. The Delphi technique is a flexible consensus building process that involves multiple rounds of questionnaires with controlled feedback provided to participants between rounds [72]. Although it has potential to build consensus, the variability in its application with respect to number of rounds and definitions for consensus, as shown in this review, has led to criticism about the methodological rigour of studies that employ this method [72]. Therefore, it is argued that the use of Delphi techniques requires additional methodological considerations and skills on the part of the researchers [72]. This may be a deterrent to using Delphi methods compared to surveys that are simpler to employ because they typically involve administering a single questionnaire without discussion or feedback from respondents.

Physiotherapist experts were consulted in the majority of competency validation steps, and physiotherapists with experience in the area of competence were consulted in the vast majority of steps that aimed to assign value to competencies. Competencies required for safe and effective practice vary based on the context of the clinical environment [7]. Therefore, the researchers likely sought input from stakeholders with relevant knowledge to enhance the applicability and accuracy of the findings to the context of interest.

There was a paucity of studies that consulted with patients and families regarding competencies for physiotherapists. Delphi technique literature has shown that the selection of stakeholders impacts research results because of differences in experience and perspectives [73]. The implications of this are two-fold. Firstly, it suggests that outcomes may have differed if other stakeholders had been involved and this might be an area for future research. Secondly, identifying definitive or absolute knowledge, skills and attitudes required for safe and effective practice may be unrealistic regardless of methodological approach. This is not necessarily problematic because competent practitioners make autonomous decisions based on the complexity of the situation [74]. To impose finite competencies could restrict safe and effective practice.

### Implications for other healthcare professions

To our knowledge, a review of this nature has not been conducted for healthcare professions other than physiotherapy. However, many healthcare professions including medicine, nursing, and occupational therapy rely on the identification of competencies to inform practice and entry-level and continuing education [17, 75]. Given the methodological variability noted for developing competency frameworks and models [17], it is unclear whether the findings from this review apply to these other professions. The novel contribution of the systematic classifications of methodological step objectives and stakeholders consulted can inform the analysis of methodological approaches for identifying competencies for other healthcare professions.

## Research gaps and future directions

1. The majority of research has focussed on established areas of physiotherapy practice such as orthopaedics. There is a gap in the literature related to competencies for emerging areas of practice, including pelvic health [15] and disaster relief [16].
2. A variety of rating scales have been used to assign value to competencies, demonstrating a lack of consistency in the literature that should be addressed in the future.
3. Few studies included patient or family perspectives, which should be considered in future research.
4. This scoping review can act as a first step towards determining ideal methodological approach(es) to competency identification through expert consensus.

## Limitations

This study did not include non-empirical publications such as perspective pieces or professional reports. This is a limitation in terms of synthesizing the nature of competencies that have been identified but does not affect the synthesis of methodologies used in the peer-reviewed empirical literature. Studies that aimed to develop, describe, or evaluate competency-based curricula, models or frameworks, or assessment tools were excluded as they were judged to be outside the scope and resources for this project. However, the authors recognize the close relationship between identifying competencies and these research aims. Nevertheless, the findings from this study may enhance future research in those areas by contributing valuable insight into the competency identification component of that research. Similarly, we excluded studies that focused on inter-professional competencies, which allowed for a more direct comparison of physiotherapy-specific research. Only one researcher was involved in the data abstraction process but the potential impact on the accuracy of the results was mitigated by using a data abstraction piloting process, abstracting text verbatim, and through the development of a codebook in the data analysis phase. Finally, this review only included studies published in English and represented research from only six of the 125 World Physiotherapy member countries [76]. The low global representation may warrant a similar review that includes research published in additional languages.

## Conclusion

This scoping review synthesized the nature of professional physiotherapy competencies and the methodologies used to identify competencies from empirical research. The results may provide methodological guidance to stakeholders interested in identifying competencies for entry-level, practicing, and specialist physiotherapists based on resources available to them.

## Supplementary Information

Below is the link to the electronic supplementary material.

**Additional file 1**. MEDLINE Search Strategy.

## Data Availability

The datasets generated during and/or analysed during the current review are available from the corresponding author on reasonable request.

## Code availability

Not applicaple.
