# Supplementary material for: Methodological approaches for identifying competencies for the physiotherapy profession: a scoping review
Source: Discov Educ. 2022 Jun 28;1(1):9. doi: 10.1007/s44217-022-00008-9 (PMC9258964; doi:10.1007/s44217-022-00008-9)
Supplement: Supplementary file 1 — Additional file 1. MEDLINE Search Strategy. [file 44217_2022_8_MOESM1_ESM.docx]

**Supplementary File 1.** MEDLINE Search Strategy

| 1 | Attitude of Health Personnel/ | 121249 |
| --- | --- | --- |
| 2 | Clinical Competence/ | 92822 |
| 3 | Professional Competence/ | 24233 |
| 4 | Health Knowledge, Attitudes, Practice/ | 110572 |
| 5 | Competency-Based Education/ | 3961 |
| 6 | competen*.tw,kf. | 122400 |
| 7 | milestone*.tw,kf. | 12569 |
| 8 | "entrustable professional activit*".tw,kf. | 458 |
| 9 | capabilit*.tw,kf. | 159742 |
| 10 | (knowledg* adj3 (attitude* or skill*)).tw,kf. | 41612 |
| 11 | 1 or 2 or 3 or 4 or 5 or 6 or 7 or 8 or 9 or 10 | 600219 |
| 12 | Physical Therapists/ | 1818 |
| 13 | Physical Therapy Specialty/ | 2823 |
| 14 | physiotherap*.tw,kf. | 26066 |
| 15 | (physical adj3 therap*).tw,kf. | 28591 |
| 16 | 12 or 13 or 14 or 15 | 53611 |
| 17 | 11 and 16 | 3770 |
